# Evaluation of Selenite Effects on Selenoproteins and Cytokinome in Human Hepatoma Cell Lines

**DOI:** 10.3390/molecules18032549

**Published:** 2013-02-26

**Authors:** Fabiola Rusolo, Biagio Pucci, Giovanni Colonna, Francesca Capone, Eliana Guerriero, Maria Rita Milone, Melissa Nazzaro, Maria Grazia Volpe, Gianni Di Bernardo, Giuseppe Castello, Susan Costantini

**Affiliations:** 1 Cancer Research Center, “Pascale Foundation” National Cancer Institute, Mercogliano (AV) 83013, Italy; 2 Biochemistry, Biophysic and General Pathology Department, Second University of Naples, Naples 80138, Italy; 3 Institute of Food Sciences—CNR, Avellino 83100, Italy; 4 Department of Experimental Medicine, Section of Biotechnology and Molecular Biology, Faculty of Medicine, Second University of Naples, Naples 80138, Italy

**Keywords:** selenite, SELENBP1, SELK, GPX1, HepG2, Huh7, hepatocarcinoma, cytokines, interactomics, inflammation

## Abstract

The need to explore new alternative therapeutic strategies and chemoprevention methods for hepatocellular carcinoma is growing significantly. Selenium is a trace element that plays a critical role in physiological processes, and is used in cancer chemoprevention. The aim of this work was to test *in vitro* the effect of sodium selenite on the human hepatoma cell lines, HepG2 and Huh7, to assess its effect on the expression of GPX1, SELK and SELENBP1 and also to evaluate its action on inflammation determinants such as cytokines. Our results show that: (i) the increase observed for the GPX1 and SELK expression is correlated with an increase in the sodium selenite concentration, also evidencing an inverse association between the levels of these two proteins and SELENBP1; (ii) the selenium concentrations evaluated in protein extracts increase in proportional way with the selenite concentrations used in the treatment, suggesting that other selenoproteins can also be modulated and should be evaluated in further studies, and (iii) some cytokines, VEGF and three pro-inflammatory cytokines, *i.e.*, IL-6, IL-8, and IL-17, decreased with an increasing selenite concentration. Finally, interactomic studies show that GPX1 and SELK, and the four pro-inflammatory cytokines are functionally correlated evidencing a putative anti-inflammatory role for the selenite.

## 1. Introduction

Selenium is a trace element for which no direct indication of its being essential in human nutrition was found until 1979 [1]. In the same year, the existence of a correlation between the low concentration of selenium in the geographical area of Keshan in China and the pathology, known as Keshan disease (due to dietary deficiency of selenium), was identified by a research group in this country [1]. Selenium is stored in human tissues in varying amounts: 30% of tissue selenium is found in the liver, 15% in the kidney, 30% in muscle, 10% in the plasma and the remaining 15% throughout the other organs [1]. Selenium is also found in trace quantities in a number of dietary agents such as dairy products, meat products, poultry, fish, fruits, vegetables and cereals, rendering it part of the food chain [2], furthermore, it is considered a significant antioxidant and precursor of the antioxidant enzyme known as “glutathione peroxidase” (GPX), which protects cells from free radical damage in a number of neuronal and neuromuscular disorders, such as stroke and cerebrovascular disease, Alzheimer’s disease, Parkinson’s disease, familial amyotrophic lateral sclerosis and Duchenne muscular dystrophy [2]. The evidence linking a lack of selenium with cancer is found in epidemiological and clinical studies where the low dietary selenium levels have become an accurate way of predicting future cancer rates [3] and, in particular, it has been shown that selenium supplementation led to a 50% reduction in cancer mortality [3]. In general, the mean content of selenium in serum from patients with various types of cancer was lower than that of the control groups. Therefore, the correlation between decreased levels of selenium and increased DNA damage and increased oxidative stress further indicates the significance of this trace element [4].

Many studies, including geographic, pre-clinical, animal, prospective as well as intervention studies, have shown selenium to be involved in gastrointestinal and liver cancers [5,6], and have suggested its putative role in their progression and, subsequently, metastasis prevention [7]. A significant positive correlation between plasma selenium levels and liver cancer was also found. Some epidemiological studies showed significantly lower serum, plasma and liver selenium levels in patients with liver diseases, such as chronic hepatitis and cirrhosis, and different grades of hepatocellular injury, compared to healthy control groups [8,9,10,11,12,13]. Hepatocellular carcinoma (HCC) develops in the liver with severe impairment of cellular antioxidant systems and some oxidative stress biomarkers for this cancer are reported in literature [14]. In particular, oxidative stress and ERK1/2 phosphorylation is reported as predictors of outcome in HCC patients treated with sorafenib plus octreotide LAR [15,16].

HCC is a major health problem worldwide, being the fifth most common malignancy in males and the eighth in females and the third most common cause of cancer-related mortality in the World. The incidence of HCC is on the increase, with marked variations among geographic regions and racial and ethnic groups, relative to the exposure to documented environmental risk factors [17]. In particular, Southern Italy has the highest rates of HCC in Europe [18], and recently it has been reported that BRAF and PIK3CA genes are somatically mutated in HCC patients of South Italy [19]. Since it has been demonstrated that selenium binding protein-1 (SELENBP1) is a protein able to incorporate exogenously administered radioactive (75Se)-sodium selenite in the liver *in vivo* [20,21], our group has recently evaluated the expression of SELENBP1 and selenium in tissue samples of HCC patients [22,23]. These studies provided evidence that this protein, as well as selenium, is down-regulated in the liver tissue of HCC patients and that its gradual loss is associated with an increased malignant grade [22,23]. On the other hand, in the literature observations related to the levels of GPX1, in which the selenium is present as selenocysteine, are contradictory, because some papers emphasized that GPX levels are increased in HCC patients, but others stated that they were reduced according to the grade of the HCC [24,25]. Moreover, selenoprotein K (SelK) is a novel endoplasmic reticulum (ER) resident protein, the biological function of which has been little characterized, indeed, a recent study showed that in HepG2 cells it was regulated by the two ER stress agents, tunicamycin and β-mercaptoethanol, and its gene silencing could significantly aggravate HepG2 cell death and apoptosis induced by the ER stress agent [26].

In the present study, we have analysed: (i) the GPX1, SELK and SELENBP1 expression by western blotting and the selenium concentrations by atomic absorption spectrometry in HepG2 and Huh7 cells after stimulation with increasing sodium selenite concentrations to understand the effect of the selenite on the protein expression, and (ii) the effect of selenite on the cytokinome of these cell lines by a multiplex biometric ELISA-based immunoassay, and have found a functional correlation among these molecules by an interactomic analysis.

## 2. Results and Discussion

### 2.1. Evaluation of SELENBP1, SELK and GPX1 Expression in HepG2 and Huh7 Cells

**Figure 1:**
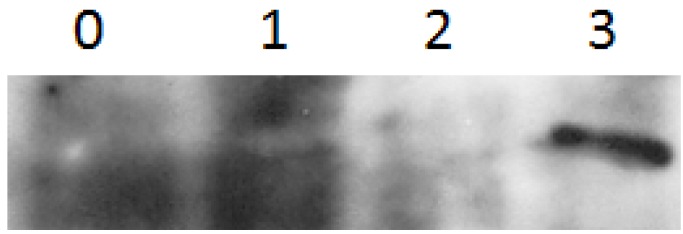
SELENBP1 protein expression analysis by western blot in the untreated HepG2 cells (1), in HepG2 cells after 24hrs of treatment with sodium selenite at the indicated doses, 0.25 mM (2) and 1mM (3), and in untreated HeLa cells (4).

We evaluated the SELENBP1, SELK and GPX1 protein expression in HepG2 and Huh7 after 24 h stimulation with different concentrations of selenite (0.25, 0.5 and 1 µM) compared to untreated cells. In untreated HepG2 and Huh7 cells SELENBP1 is not expressed (Figure 1), in agreement with a recent article that evidenced very marginal levels of this protein in HepG2 and HuH7 as well as in other non metastatic HCC cell lines [27].

**Figure 2:**
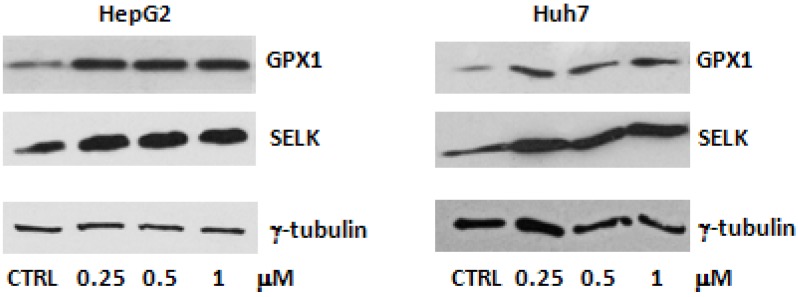
SELK and GPX1 protein expression analysis by Western blot in the HepG2 and Huh7 cell lines after 24 h of treatment with sodium selenite at the indicated doses. γ-tubulin was used as loading control.

We used as positive control for anti SELENBP1 antibody HeLa cells, known to express high levels of this protein [28]. However, even if SELK and GPX1 are expressed in the untreated HepG2 and Huh7 cells, the expression of SELK is less than that of GPX1 (Figure 2).

**Figure 3:**
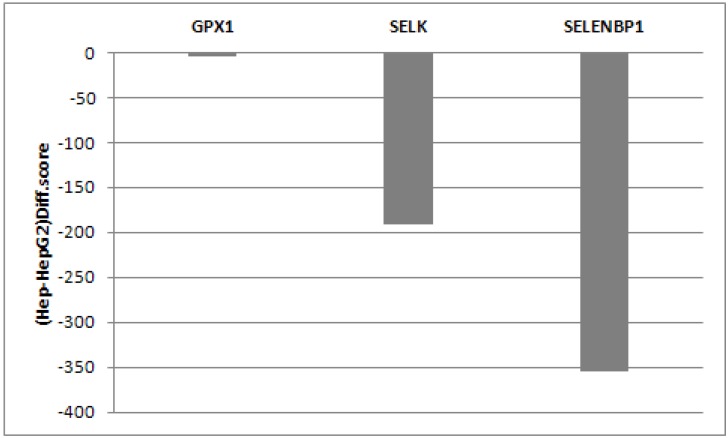
Difference score (Diff.score) between hepatocytes and HepG2 cells.

This is in agreement with the gene expression analysis which evidenced that SELK was found down-regulated in HepG2 when compared to normal hepatocytes, whereas the expression of GPX1 was similar in normal and cancerous cells (Figure 3). Moreover the treatment with selenite in both cell lines produces an increase in the 11 KDa SELK as well as 23 KDa GPX1 protein expression already at low doses of selenite, and levels of both proteins were found constant, independently from used concentrations (Figure 2). In the meantime, the 40 KDa SELENBP1 protein was not appreciable.

Our results agree with a previous study that investigated the biological effects of selenium in human hepatoma Hep3B cells evidencing that selenium supplementation restored the GPX activity when these cells are kept in selenium-deficient media [29]. Similar results were also shown in another study in which increased GPX1 levels resulted in the reduction of SELENBP1 in human colorectal as well as breast cancer cells [30]. This effect was demonstrated to be due, at least in part, to the inhibition of SELENBP1 transcription. However, even if little is known about the regulation of SELENBP1 transcription, it is possible that SELENBP1 expression is dependent on reactive oxygen-responsive transcriptional elements, and the reduction in peroxides that is expected to be achieved with increased GPX1 activity can result in the attenuation of transcription [30].

However, in the literature we did not find any information about the expression and modulation of SELK in liver cancer cells after selenium up-take and, hence, our data evidence for the first time a positive correlation between this protein and selenite in hepatic cells.

### 2.2. Evaluation of Selenium Concentrations

Since SELK and GPX1 protein expression increase already at low doses of selenite and, then, remained constant, we evaluated by atomic absorption spectrometry the concentration of selenium in the same protein extracts from HepG2 and Huh7 after stimulation with different concentrations of selenite compared to untreated cells in order to understand if the cells absorbed only the selenium present in SELK and GPX1 or also other amounts.

**Figure 4:**
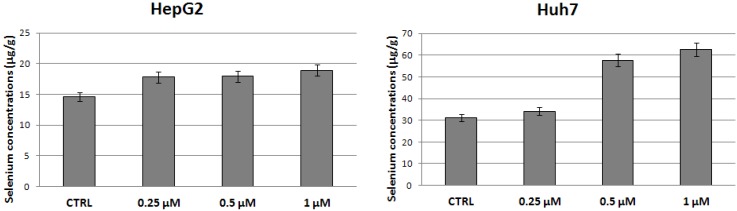
Selenium concentrations (μg/g) in protein extracts of HepG2 and Huh7 after 24 h of treatment with sodium selenite at the indicated doses.

As shown in Figure 4, the selenium concentration increases with increasing sodium selenite concentrations (with *p* < 0.05) in both cell lines even if in different amount and through different trend. This has evidenced that also other selenoproteins are modulated from cellular treatment with selenite.

Moreover, we can suggest that the different concentrations evaluated in HepG2 and Huh7 could depend from the fact that Huh7 cells are more undifferentiated respect to HepG2. In fact, recent studies have shown that the Huh7 cell line is associated with low expression of cytokeratin 8/18 (CK8/18), while HepG2 cell line is correlated with high expression of CK8/18 being usually expressed in normal hepatocytes [31]. In addition, HepG2 cells express p53 in their native form while Huh7 cells constitutively express the mutated form of the same protein and, therefore, are characterized by a more malignant phenotype [32].

### 2.3. Bio-Plex assay on HepG2 and Huh7 cells

**Figure 5:**
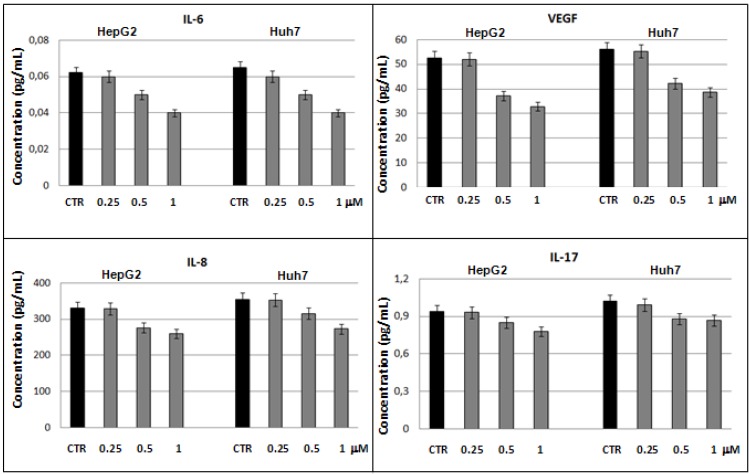
Significant cytokine levels (with *p* < 0.05) in HepG2 and Huh7 cells line after 24h of treatment with sodium selenite.

Since in HCC the constant inflammation resulted to play an important role in the transition from chronic liver disease to neoplastic process [33,34], we also studied the immuno-modulatory role of the selenite on the cytokines production in hepatoma cellular supernatants by a multiplex biometric ELISA-based immunoassay. In details, we evaluated the cytokine levels in HepG2 and Huh7 supernatants after incubation with sodium selenite at 24 h. The obtained results were compared with untreated cells used as control. These experiments showed that the levels of VEGF and three pro-inflammatory interleukins, like IL-6, IL-8, and IL-17, decreased in statistically significant way at increasing concentrations of sodium selenite (Figure 5).

Studies on HCC patients, conducted in our laboratory, showed that high levels of IL-8 and IL-6 correlated with tumor size suggesting that these two proteins could have a role during the HCC progression and can be considered as markers of tumor invasiveness [30].

In particular, IL-8 is a pro-inflammatory chemokine (CXCL8) having a strong pro-angiogenic activity in HCC patients [35] and its expression has been correlated with invasiveness and tumor metastasis because it increased significatively in the later stages of HCC [36]. Moreover, IL-17 is a pro-inflammatory interleukins that stimulates fibroblasts and epithelial and endothelial cells, macrophages and keratinocytes to produce some cytokines such as IL-6 and IL-8 and its function is essential to a subset of CD4 + T cells called T helper 17 (Th17) whose role is linked to many immune and autoimmune diseases [37].

IL-6 acts as both a pro-inflammatory and anti-inflammatory cytokine and is one of the most important mediators of the acute phase response. In cancer cells, it stimulates the secretion of VEGF being an established potent angiogenetic factor with pro-inflammatory properties [38]. Recently it has been reported that selenite inhibits the expression of VEGF and IL-6 induced by lipopolysaccharide in human prostate cancer PC3 cells via TLR4-NF-(K)B signalling blockage [39]. This evidences that the decreased levels of IL-6, IL-8, IL-17 and VEGF are certainly correlated between them. Hence, our results suggest that selenite could inhibit the tumor invasiveness promoting cellular regression and have an anti-inflammatory effect according to our studies on lipoic acid and caffeic acid which evidenced that these molecules reduce the expression of some pro-inflammatory cytokines in HepG2 and Huh7 cells [40].

### 2.4. Interactomic Studies

Specific undirect or direct physical interactions among the molecules evidenced in this paper and biochemically different in their structure have been assessed also by an interactomic analysis. In general, interacting molecules form molecular interaction networks that are classified by the nature of the compounds involved.

**Figure 6:**
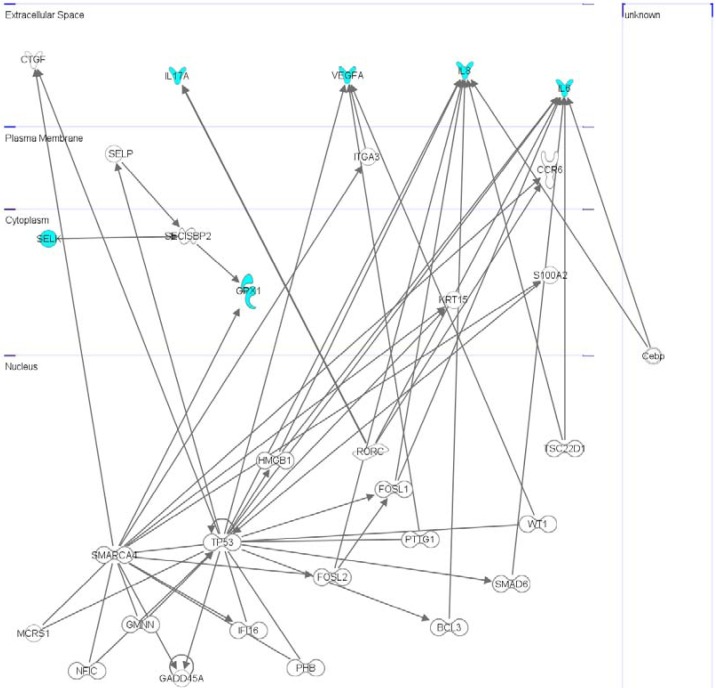
Interactomic analysis by Ingenuity Pathway Analysis (IPA) of significant molecules. The interactome shows the close functional association between SELK, GPX1 and significant cytokines, (evidenced with cyan symbols) as well as the paths in whichother functionally relevant moleculesare also involved (evidenced with white symbols).

The four significant cytokines and the two seleno-proteins, SELK and GPX1, were analysed by Ingenuity Pathway Analysis 7.1 (Ingenuity Systems, Inc., Redwood City, CA, USA). The network is generated by associated functions and data mining from experimental data reported in literature, and our molecules have been found involved into a network named “Tissue Development, Gene expression, Cell Death and Survival” where IL-6, IL8, IL-17, VEGF and GPX1 are connected with two hub genes, correlated between them [41] such as SMARCA4 and encoding chromatin remodeling complex components and TP53 (tumor p53) that responds to different types of cellular stresses to regulate target genes by inducing cell cycle arrest, apoptosis, senescence, DNA repair, or metabolic changes (Figure 6).

Their related proteins are known to create a protein complex. In details, SMARCA4 is connected with GPX1 and VEGF via ITGA3 (integrin alpha3) whereas TP53 with VEGF, IL-6, IL-8 and SELP (selenoprotein P) that, together with SELK, interacts with SECISBP2 that functions as a SECIS (sec insertion sequence) binding protein useful for the incorporation of selenocysteine also into GPX1 [42]. On the other hand, IL-17 is correlated with IL-6 through RORC that decrease the expression of IL-17 and IL-6 in differentiating human naïve T lymphocytes [43]. The power of the interactomic studies depend on the fact that the molecular members found in a network are also member of functionally different sub-nets thus they transfer to the more general network also the different cellular and biochemical functions related to each specific sub-net [44]. In conclusion, recalling that TP53 is modulated by NF-κB [45], our network analysis confirms that the four cytokines are strictly correlated between them, and the decrease of their levels can induce NF-κB inactivation and, hence, an inflammation decrease in the hepatoma cells clearly assessing a reliable putative anti-inflammatory role for the selenite.

## 3. Experimental

### 3.1. Cell Culture

Human hepatoma cell lines (HepG2 and Huh7) were kept in culture and expanded at 37 °C in a humidified atmosphere of 5% CO_2_ in culture medium DMEM (Dulbecco's Modified Eagle’s Medium, Lonza, Verviers, Belgium), supplemented with FBS (Invitrogen, Camarillo, CA, USA) at 10%, Penicillin/Streptomycin 100x (Euroclone, Devon, UK), Glutamax 100x (Invitrogen) and non-essential amino acids 100x (Invitrogen). Phosphate buffer (PBS phosphate buffered saline Ca^2+^ and Mg^2+^ free) and trypsin (Ca^2+^ and Mg^2+^ free) were supplied by Euroclone.

The cells (2 × 104) were seeded in 100 mm plates in 8 mL of culture medium, and left to grow for 24 h at 37 °C to allow adhesion. Then, the cells were treated with sodium selenite, dissolved in H2O, at the following concentrations 0.25 µM, 0.5 µM and 1 µM and incubated for 24 h. The experiments were performed in duplicate and repeated for three times. However, the concentrations of sodium selenite used in this study were chosen concerning that human physiological concentration of selenium is less than 3 µM [46].

### 3.2. Protein Extraction and Western Blot Analyses

HepG2 and Huh7 cells were washed once in cold phosphate buffered saline (PBS) and lysed in a lysis buffer containing 20 mM Tris HCl pH 7,5, 150 mM NaCl, 1mM EDTA and NP40 after 24 h of treatment with 0.25, 0.5 and 1 µM of sodium selenite. The lysis buffer was complemented with protease inhibitor cocktail tablets (Roche Applied Science, Penzberg, Germany), diluted in H_2_O to obtain a stock solution 7X concentrated, and with phosphatase inhibitor cocktail tablets (Roche Applied Science), diluted in H_2_O to obtain a 100× concentrated stock solution. The lysates were clarified by centrifugation at 13,000 rpm for 15 min. Protein concentrations were estimated by a BioRad assay (Bio-Rad Laboratories, Hercules, CA, USA), based on the method of Bradford, that is a simple and accurate procedure for determining concentration of solubilized protein. Then the proteins were boiled for 5 min before electrophoresis in Laemmli Sample buffer (Bio-Rad) containing 62.5 mM Tris-HCl pH 6.8, 2% sodium dodecyl sulphate (SDS), 25% glycerol, 0,01% bromophenol blue complemented with 10% β-mercaptoethanol. 60 μg of proteins were subjected to SDS–polyacrylamide gel electrophoresis (PAGE) using 12% acrylamide concentrated gels under reducing condition. After electrophoresis, proteins were transferred to nitrocellulose membranes (Amersham Hyperfilm MP, High performance autoradiography, GE Healthcare, Hertfordshire, UK); complete transfers were assessed using prestained protein standards (Fermentas, Milano, Italy). After blocking with Tris-buffered saline 5% non fat dry milk (Bio-Rad), membranes were incubated ON at 4 °C in shaking with the Goat anti GPX1 antibody (R&D Systems, Minneapolis, MN, USA), with the rabbit anti SELK antibody (ABCAM, Cambridge), and with the rabbit anti SELENBP1 antibody (ABCAM, Cambridge, UK) diluted 1:500, 1:500 and 1:1,000 overnight at 4 °C, respectively, and then incubated with the horseradish peroxidase conjugated secondary antibody (1:3,000) for 60 min at room temperature; the reaction was detected with a Western blotting detection system (ECL; Amersham Biosciences, Little Chalfont, UK). To ascertain that equal amounts of protein were loaded, membranes were incubated with antibodies against the γ-tubulin protein (1:500) (Santa Cruz Biotechnology, Santa Cruz, CA, USA).

### 3.3. Atomic Absorption Spectrometer Studies

Nitric acid 67% and ultrapure water were from Sigma-Aldrich (Steinheim, Germany). Selenium standard (1,000 ppm) was from Carlo Erba Reagents (Milan, Italy). The sample preparation for quantitative determination of selenium is performed according to the method reported by O’Neill *et al*. [47], with some modifications. The samples were sonicated, dissolved in 1% aqueous HNO_3_, and kept at 50 °C overnight. The selenium concentration was determined by graphite furnace atomic absorption spectroscopy on a Varian SpectrAA200 (Victoria, Australia) spectrometer with Zeeman background correction. The quantitative determinations were carried out by a calibration curve using selenium standard solutions (5–20 ppb). Digested samples were diluted with ultra pure water to bring the selenium concentration within the calibration range. The furnace settings were as follows: for drying, ramp to 85 °C (5 s), ramp to 95 °C (40 s), and ramp to 120 °C (10 s); for washing, ramp to 1,000 °C (5 s) and hold at 1,000 °C (3 s); for atomization, ramp to 2,600 °C (0.8 s, read signal), hold at 2,600 °C (2 s, read signal), and hold at 2,600 °C (2 s, tube clean). The absorbance was determined at 196 nm, the slit was 1.0 nm. Results were expressed as µg selenium per g of protein. Data presented are the average of two measurements.

### 3.4. Bio-Plex Assay

In our approach, the levels of a panel of numerous cytokines, chemokines, and growth factors were evaluated at the same time by BioPlex assay. The simultaneous quantitative determination of a large panel of cytokines, able to report the correct ratios and dynamics between highly and poorly represented molecules, has emerged as an accurate, simple, specific, noninvasive, reproducible and less expensive method [33,34,40]. The multiplex biometric ELISA-based immunoassay, containing dyed microspheres conjugated with a monoclonal antibody specific for a target protein was used, according to the manufacturer’s instructions (Bio-Plex Bio-Rad), to evaluate the concentrations of different cytokines by Human Cytokine 27-Plex Panel after 24 h of incubation with sodium selenite in HepG2 and Huh7 supernatants. In particular, the following cytokines were evaluated: IL-1β, IL-1ra, IL-2, IL-4, IL-5, IL-6, IL-7, IL-8, IL-9, IL-10, IL-12 (p70), IL-13, IL-15, IL-17, eotaxin (CCL11), basic FGF, G-CSF, GM-CSF, IFN-γ, CXCL10, MCP-1, MIP-1α, MIP-1β, PDGF-ββ, RANTES, TNF-α and VEGF. Each experiment was performed in duplicate as previously described [33,34,40]. Protein concentrations were determined using a Bio-Plex array reader (Luminex, Austin, TX, USA) that quantitates multiplex immunoassays in a 96-well format with very small fluid volumes. The analyte concentration was calculated using a standard curve, with software provided by the manufacturer (Bio-Plex Manager Software).

### 3.5. Statistical Analysis

The cytokines concentrations evaluated in HepG2 and Huh7 supernatants after 24 of incubation with sodium selenite were compared by T-test. Values of *p* < 0.05 were considered to be statistically significant. The statistical program Prism 4 (GraphPad Software, San Diego, CA, USA) was used.

## 4. Conclusions

HCC treatment with conventional chemotherapeutic agents is inefficient, due to several side effects linked to impaired organ function typical of liver diseases. Therefore, it needs to explore possible chemo-preventive alternative and/or therapeutic strategies. In fact, the use of dietary antioxidants and micronutrients has been recently proposed for successful HCC management [48]. The goal of our research was to investigate whether the treatment with selenite shows functional effects on GPX1, SELK and SELENBP1, which belong to two different classes of selenium containing molecules, and on the hepatoma cell cytokinome. To accomplish this purpose, we have analyzed the expression of SELENBP1 and GPX1 in HepG2 and tested the effects of sodium selenite on these cells. Moreover, we have evaluated the cytokine concentrations in order to evaluate the pro- or anti-inflammatory effects of sodium selenite. Our data evidenced that sodium selenite induced: (a) the increase of GPX1 and SELK protein expression in both HepG2 and Huh7 cells treated with increasing selenite concentrations, and (b) the related decrease of VEGF and of three pro-inflammatory cytokines, *i.e.*, IL-6, IL-8, and IL-17. Our data were also confirmed by an interactomic analysis that showed also the functional paths in which these molecules are involved.

On the whole, the work presented herein establishes that not only in HCC there is a correlation between the selenium availability, and the cellular amount of two proteins, GPX1 and SELK, but also that sodium selenite shows an anti-inflammatory effect and might, therefore, inhibit the tumor invasiveness promoting a cellular regression also because is adequately related to the specific functional paths. However, whether any of the possible benefits or risks of selenium intake are due to consequential effects on GPX1 and SELK must be more amply demonstrated.

Moreover, since the selenium concentrations, measured by atomic absorption spectrometry, were shown to increase in proportional with those of selenite used in the treatment, we can suggest that other selenoproteins can also be modulated and should be evaluated. Therefore, our further studies will regard the proteomic evaluation of hepatoma cell lines treated with increasing amount of selenite to identify in what metabolic pathways these proteins are involved and the other possible markers involved in these processes.
